# The Southern Surgical and Gynecological Association

**Published:** 1889-12

**Authors:** 


### Society Meetings.

THE SOUTHERN SURGICAL AND GYNECOLOGICAL ASSOCIATION held a most interesting and instructive meeting at Nashville, Tenn., November 12, 13, and 14, 1889. The attendance was large and the papers, for the most part, were of a high order of merit. To have organized and put in active working order so useful a medical body in so short a time, reflects great credit upon its founders, and no one is entitled to greater praise than its accomplished and indefatigable

Secretary, Dr. W. E. B. Davis, of Birmingham, Ala. The great glory of Dr. Hunter McGuire, of Richmond, Va., a man already accustomed to the plaudits of his professional brethren, is that he was chosen to preside at this second annual meeting of this famous association, where, under his experienced and able guidance, so much good work was done. It will be no easy matter even for this young, vigorous, and progressive Association to improve upon its Nashville meeting; though it may be predicted that the next year at Atlanta, under the presidency of Dr. George J. Englemann, of St. Louis, a notable meeting will be held.

